# Transient expression of homologous hairpin RNA interferes with PVY transmission by aphids

**DOI:** 10.1186/1743-422X-5-42

**Published:** 2008-03-19

**Authors:** Marisol Vargas, Belén Martínez-García, José Ramón Díaz-Ruíz, Francisco Tenllado

**Affiliations:** 1 Departamento de Biología de Plantas, Centro de Investigaciones Biológicas, (CIB, CSIC) Campus de la Ciudad Universitaria, Av. Ramiro de Maeztu 9, 28040 Madrid, Spain

## Abstract

Hairpin RNAs have been used to confer resistance to viruses in plants through RNA silencing. However, it has not been demonstrated that RNA silencing was effective against inoculation by aphids of non-persistently transmitted viruses, the major route of plant virus spread in nature. As a proof-of-principle strategy, we made use of *Agrobacterium tumefaciens *to transiently express a hairpin RNA homologous to *Potato virus Y *(PVY) in plant tissues. A complete and specific interference with aphid transmission of PVY was achieved by inducers of RNA silencing, as demonstrated by specific siRNAs accumulation in agroinfiltrated tissues. To our knowledge, this is the first report of successful interference with non-persistent transmission of a plant virus using RNA interference.

## Findings

One of the most efficient mechanisms by which plants protect themselves from viruses is the specific RNA-dependent silencing pathway termed post-transcriptional gene silencing (PTGS). In certain circumstances, the RNA silencing machinery recognizes several features of viral infections involving the formation of double-stranded (ds) RNA and initiates a response that degrades viral RNA and eventually enables the plant to recover from virus infection [1]. This principle can be manipulated by biotechnologists to confer resistance to crops against virus diseases in several ways. Several studies have demonstrated that inverted repeat constructs encoding self-complementary RNAs (hairpin RNAs) can effectively induce RNA silencing and lead to high resistance frequencies in transgenic plants [2,3]. However, questions concerning the potential ecological risk of virus-resistant transgenic plants, including genetic flow and reversal of silencing by viral suppressors, have so far significantly limited its use [4]. As an alternative approach, we and others have previously shown that exogenously supplied dsRNA, or vectors expressing it, derived from viral sequences can specifically interfere with virus infection in non-transgenic plants [5-7]. It was proposed that the effect mediated by direct application of dsRNA onto plant surfaces concurrent to mechanical inoculation of the virus resembles the analogous phenomenon of RNA interference (RNAi) observed in animals [8,9]. The interfering dsRNA would mimic double-stranded forms of RNA produced during virus replication, triggering the initiation step of PTGS. This may lead to the production of 21 to 24 nucleotides duplexes (small interfering RNAs, siRNAs) which are incorporated into a nuclease complex responsible for the degradation of the cognate viral RNA [1]. Thus, the invading virus containing sequences homologous to the dsRNA is recognized and degraded by the plant's defence mechanism. This non-transgenic, RNAi-based approach could form the basis for the development of a new biotechnological tool aimed at protecting crops against virus diseases [9].

Aphid transmission is the main method of spread for most plant viruses in nature including members of the genus *Potyvirus*, the largest group of plant viruses. These viruses are transmitted in a non-persistent manner, a category of non-circulative transmission also known as stylet-borne [10]. Since the RNAi-based approach in non-transgenic plants has only so far been demonstrated against mechanically inoculated viruses, we sought to expand this resistance against virus inoculation by aphids. As a proof-of-principle strategy, we made use of *Agrobacterium tumefaciens *to transiently express hairpin RNA molecules in plant tissues. The *Agrobacterium*-mediated transient expression system has previously been used to deliver RNA silencing inducers and suppressors into plants [11]. We previously showed that transient expression of a hairpin RNA could block multiplication and spread of a tobamovirus delivered by mechanical inoculation in non-transgenic plants [12]. However, it is not known if transient expression of hairpin RNA could block virus transmission by aphids. In the present study, we investigated the silencing potential of inverted repeat sequences designed to generate a hairpin RNA homologous to *Potato virus Y *(PVY) for its ability to interfere with the non-persistent transmission of this virus by aphids.

**Figure 1:**
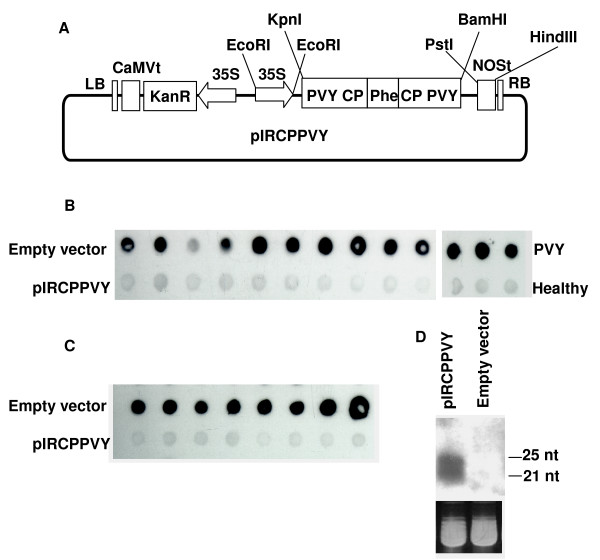
**Detection of PVY CP and PVY small interfering RNAs in *N. benthamiana *agroinfiltrated tissues.(A) **Schematic representation of pIRCPPVY used for transient expression by agroinfiltration. A cDNA fragment encoding sense and antisense PVY CP RNA sequences separated by a spacer sequence (Phe) were cloned into binary plant vector pCAMBIA2300. **(B) **Plants were agroinfiltrated with empty vector or pIRCPPVY and used after 4 days post-infiltration to inoculate PVY. Where no agroinfiltration occurred (right panel), plants were directly inoculated with PVY (PVY) or buffer (healthy). **(C) **Plants were agroinfiltrated with empty vector or pIRCPPVY and used after 4 days post-infiltration to feed viruliferous aphids (*M. persicae*). At 14 dpi., sap extracts from upper leaves of single plants were assayed by dot blot using PVY antiserum, and detected using a secondary antibody conjugated to peroxidase. **(D) **Northern blot analysis of low molecular weight RNAs shows the accumulation of siRNAs in pIRCPPVY-infiltrated leaves. Samples were taken 4 days after infiltration. The blot was hybridised with a ^32^P-labeled cDNA PVY CP probe. Equivalent loading of samples was shown by staining the gel with ethidium bromide before transfer. The mobilities of oligodeoxynucleotides of the indicated length are shown to the right.

The commercial plasmid pCAMBIA2300 (CAMBIA) was used to express *in planta *the inverted-repeat RNA corresponding to coat protein (CP) gene sequences of PVY. First, the cauliflower mosaic virus (CaMV) 35S promoter was extracted from pAVA393 [13] using *EcoR*I, and ligated to a derivative of pCAMBIA2300 in which the 3' terminator region of the nopaline synthase gene (NOSt) was inserted between *Pst*I and *Hin*dIII sites. The viral sequences were then extracted from pBKS-IRCPPVY using *Kpn*I and *BamH*I and introduced into the pCAMBIA2300 derivative to generate pIRCPPVY. To prepare pBKS-IRCPPVY, a fragment of plasmid pBSK-CPPVY [14] was amplified by polymerase chain reaction (PCR) using primers 5'-TCCTCTAGA*CACTGAAATGATGG*-3' and 5'-CTTGGTACC*GGAGAGACACTACATC*-3', corresponding to positions 8509–8523 and complementary to 9374–9390, respectively (italized), in the PVY genome [15]. An *Xba*I and *Kpn*I restriction sites (underlined) were created in both primers, respectively, to facilitate further cDNA cloning in a triple-ligation reaction: the PCR product was cut with *Xba*I-*Kpn*I; pCB278, a plasmid harbouring the phleomycin resistance gene [7], was cut with *Hind*III-*Xba*I and both fragments were ligated to the *Kpn*I-*Hind*III cut pBSK-CPPVY to obtain pBKS-IRCPPVY. Sequencing confirmed the correct ligation of the three components. pBKS-IRCPPVY then incorporates a bacterial gene as a spacer sequence between the sense and the antisense orientations of the PVY CP sequence. We took advantage of the positive selection conferred by this prokaryotic resistance gene to reduce the risk of intramolecular homologous recombination in bacteria. Upon transcription, RNA from pIRCPPVY is intended to fold into a stem-loop structure consisting of 881 bp of PVY dsRNA and approximately a 500 bp spacer sequence (IRCPPVY, Fig. 1A). For comparison, one additional construct containing inverted repeat sequences corresponding to the *Pepper mild mottle virus *(PMMoV) polymerase gene (IR54PMMoV) [7] was used. An empty pCAMBIA2300 was also used as a negative control. The binary plant vectors were introduced into *Agrobacterium tumefaciens *GV2260 by triparental mating and the infiltration of plant tissues was performed essentially as described by Tenllado et al. [7].

We determined first whether transient IRCPPVY expression could trigger an antiviral response in plants against mechanically inoculated PVY. *Nicotiana benthamiana *plants were infiltrated with *A. tumefaciens *cultures carrying IRCPPVY or the empty vector. At 4 days post-infiltration, plants were challenge-inoculated by applying 20 μl of PVY sap inoculum (1/10 w/v) on the infiltrated leaves dusted with Carburundum. Plants were kept in growth chamber with a 16 h light/8 h dark cycle at 25°C. By 7 days post-inoculation (dpi), mosaic symptoms were displayed by PVY-inoculated plants that had been infiltrated with *Agrobacterium *carrying the empty vector. In contrast, inoculated plants that had been infiltrated with IRCPPVY remained symptomless throughout the entire testing period (2 months). Analyses of viral CP accumulation were performed by dot blot analysis (Fig. 1B). Plant extracts were prepared by homogenizing leaf tissue in phosphate-buffered saline, pH 7.4 (PBS) (5 ml/g) and clarified by centrifugation at 10,000 rpm for 5 min. 10 μl of extracts diluted in 200 μl of PBS were applied to polyvinyledene difluoride (PVDF) membranes (Amersham) using a Bio-dot SF microfiltration apparatus (BioRad), and washed twice with PBS. The sensitivity was increased by incubating the membranes in acetone for 1 min, prior to reaction with the specific antiserum. A specific rabbit antiserum against PVY [14] was used at 1:1,000 in PBS containing 5% nonfat milk and 0.05% Tween 20. Blots were washed with PBS and incubated with peroxidase-conjugated goat antirabbit IgG (GARPO) diluted 1:15,000 in PBS containing 5% nonfat milk for 1 h at room temperature. Blots were washed twice with PBS and enzyme activity was detected with an enhanced chemiluminescence kit (ECL, Amersham). Dot blot analysis of total soluble proteins extracted from systemic leaves at 14 dpi showed that PVY CP accumulated in plants that had been infiltrated with the empty vector at comparable levels to PVY-inoculated plants that had not been infiltrated with *Agrobacterium*. In agreement with the lack of symptoms, PVY CP was not detected in the upper leaves of plants infiltrated with IRCPPVY in any of the ten plants used in the assay. These results suggest that transient expression of PVY hairpin RNA is competent to trigger an antiviral response against mechanically transmitted virus.

In a set of new experiments, leaves of *N. benthamiana *plants were agroinfiltrated with cultures of *Agrobacterium *carrying IRCPPVY, IR54PMMoV or the empty vector constructs. At 4 days post-infiltration, agroinfiltrated leaves were used in plant-to-plant transmission tests. Groups of apterous mature aphids from a *Myzus persicae *(Sulzer) clone were collected, starved for a period of 2–3 h and allowed to probe for 5–10 min on leaves of PVY-infected *N. benthamiana *plants. Aphids (10–15 per plant) were released onto an agroinfiltrated leaf covered with a plastic bag at 4 days post-infiltration for inoculation over a 2 h period before spraying with pirimicarb at 0.05% (w/v). Plants were transferred to the growth chamber for observation. The results (Table 1) showed that transiently expressed IRCPPVY was able to block transmission of PVY (0% transmission rate), while aphids fed on plants infiltrated with the empty vector transmited the virus at high efficiency (100% transmission rate). Sixteen out of sixteen plants infiltrated with *Agrobacterium *containing the empty vector displayed disease symptoms in upper leaves at 7 dpi, whereas all plants (16 plants in 2 independent experiments) that had been agroinfiltrated with the IRCPPVY construct were free of symptoms until their life cycles were completed. Dot blot analysis confirmed the visual observations of the accumulation of PVY CP in upper leaves at 14 dpi (Fig. 1C). PVY CP was not detectable in plants infiltrated with IRCPPVY, whereas viral CP was abundant in plants infiltrated with *Agrobacterium *containing the empty vector. The interfering activity on PVY transmission exhibited by transiently expressed IRCPPVY could reflect any kind of unspecific, defence response of plants elicited by hairpin RNA sequences that could somehow block the transmission process. However, *Agrobacterium*-mediated expression of a hairpin RNA derived from a heterologous virus, IR54PMMoV, had no detrimental effect on PVY transmission by aphids (Table 1). These results suggest that interference with virus transmission by inverted repeat sequences was sequence-specific and likely due to the activation of RNA silencing.

**Table 1:** Interference with PVY transmission by *Agrobacterium tumefaciens*-mediated transient expression of IRCPPVY.

Agroinfiltrated^a^	Experiments	Total^b^	Percentage transmission
Empty vector	2	16/16	100
pIRCPPVY	2	0/16	0
pIR54KPMMoV	1	8/8	100

^a ^*N. benthamiana *leaves were agroinfiltrated with the indicated constructs, and used after 4 days post-agroinfiltration to feed viruliferous aphids (*M. persicae*).

^b ^Plant-to-plant transmission experiments were performed, using 10–15 aphids per plant. Pooled transmission rates are indicated as number of infected plants/number of test plants

To further test whether interference with aphid transmission conferred by transient expression of hairpin RNA could be attributable to PTGS, the low molecular weight RNA fraction was extracted from non-inoculated, *Agrobacterium*-infiltrated leaves and analysed by Northern blot hybridisation as described [16]. A ^32^P-labeled probe specific for the PVY CP sequence cloned in pBSK-CPPVY was produced by random priming. siRNA species characteristic of PTGS were detected in plants that had been infiltrated four days before with IRCPPVY but were absent in empty vector-infiltrated plants (Fig. 1D).

We have demonstrated that transient expression of an antiviral hairpin RNA by *A. tumefaciens *results in resistance to PVY transmission by aphids. Several studies have indicated that inverted repeat constructs of transgenes can effectively induce RNA silencing and protect plants against viruses, including PVY, transmitted mechanically [2,3]. However, it was not formally demonstrated that silencing triggered by hairpin RNA was effective against inoculation by aphids of non-persistently transmitted viruses, the major route of virus spread in nature. Here, it is shown that a complete and specific interference with aphid transmission of PVY can be achieved by inducers of RNA silencing, as suggested by specific siRNAs accumulation in agroinfiltrated tissues. The available evidence indicates that *Agrobaterum-*mediated expression of constructs driven by the 35S promoter occurs in virtually every *N. benthamiana *cell, including the epidermal and mesophyll cell layers where inoculation of non-persistently transmitted viruses by aphids take place [17]. As a result, PVY hairpin RNA is converted into siRNAs that guide sequence-specific cleavage of the incoming, homologous viral RNA. Wang et al. [18] reported resistance in barley transgenic plants expressing *Barley Yellow Dwarf Virus *(BYDV)-hairpin RNA to the transmission of this virus by aphids. BYDV is a persistently transmitted, circulative virus and thus represents a different transmission mechanism, with distinctive impact on plant virus epidemiology, to that of PVY [10]. Since inoculation by aphids of BYDV and potyviruses occurs in different cell layers (phloem vs. epidermis), features of the silencing response required to achieve interference with transmission might differ.

Once having established the principle that RNAi is a highly efficient approach to interfere with the non-persistent transmission of plant virus by aphids, we envision extending the non-transgenic, RNAi-based approach to confer protection against aphid-borne viruses by directly delivering dsRNA to plants [9]. Several expression systems have been developed for the production of dsRNA in bacteria that could scale-up and speed the cost-effective production of viral-derived interfering molecules [7,19]. In order to enhance the therapeutic efficacy of RNAi against aphid transmission of viruses, new and better strategies for delivery of dsRNA to plant tissues have to be developed. Beyond this therapeutic application for plant protection, hairpin-dependent interference with virus transmission by transiently expressed constructs provides a potential tool to further dissect the molecular mechanisms of aphid transmission. Expression of hairpin RNAs driven by tissue-specific regulatory sequences could be employed to abort virus multiplication at specific plant cell layers and study their consequences for non-persistent and other types of transmission processes.

## Competing interests

The author(s) declare that they have no competing interests.

## Authors' contributions

JRDR, FT; Design and conception of study. MV;cloning cDNA constructs and dot blot analysis. MV, BM-G; transmission assays. FT; manuscript preparation. All authors read and approved the final manuscript.
